# Can strength training or tai ji quan training reduce frailty in postmenopausal women treated with chemotherapy? A secondary data analysis of the GET FIT trial

**DOI:** 10.21203/rs.3.rs-3425168/v1

**Published:** 2023-10-17

**Authors:** Kerri M. Winters-Stone, Sydnee Stoyles, Nathan Dieckmann, Elizabeth Eckstrom, Shiuh-Wen Luoh, Fay Horak, Eric J. Roeland, Fuzhong Li

**Affiliations:** Oregon Health & Science University; Oregon Health & Science University; Oregon Health & Science University; Oregon Health & Science University; Portland Veteran’s Affairs Medical Center; Oregon Health & Science University; Oregon Health & Science University; Oregon Research Institute

**Keywords:** aging, physical activity, exercise, neoplasm, physical functioning

## Abstract

**Purpose:**

To determine whether strength training or tai ji quan can reduce frailty in older, postmenopausal women treated with chemotherapy for cancer.

**Methods:**

We conducted a secondary data analysis from a 3-arm, single-blind, randomized controlled trial where older (50+ years), postmenopausal women cancer survivors were randomized to supervised group exercise programs: tai ji quan, strength training, or stretching control for 6 months. We assessed frailty using a 4-criteria model consisting of weakness, fatigue, inactivity, and slowness. Using logistic regression, we determined whether the frailty phenotype (pre-frailty or frailty) decreased post-intervention, how many and which frailty criteria decreased, and what characteristics identified women most likely to reduce frailty.

**Results:**

Data from 386 women who completed baseline and 6-month testing were used (mean age of 62.0 ± 6.4 years). The odds of improving overall frailty phenotype over 6 months was significantly higher in the strength training group compared to controls (OR [95%CI]: 1.86 [1.09, 3.17]), but not for for tai ji quan (1.44 [0.84, 2.50]). Both strength training (OR 1.99 [1.10, 3.65]) and tai ji quan (OR 2.10 [1.16, 3.84]) led to significantly higher odds of reducing ≥1 frailty criterion compared to controls. Strength training led to a three-fold reduction in inactivity (p <0.01), and tai ji quan to a two-fold reduction in fatigue (p=0.08) versus control. Higher baseline BMI, comorbidity score, and frailty status characterized women more likely to reduce frailty than other women.

**Conclusions:**

Strength training appears superior to tai ji quan and stretching with respect to reducing overall frailty phenotype among postmenopausal women treated with chemotherapy for cancer, but tai ji quan favorably impacted the number of frailty criteria.

**Implications for Cancer Survivors:**

Supervised, group exercise training that emphasizes strength training and/or tai ji quan may help combat accelerated aging and reduce frailty after cancer treatment.

## Introduction

The growing number of older cancer survivors will pose a significant challenge for the healthcare system because of the combined effects of cancer treatment and aging on the development of comorbid disease, disability, and fall-related injuries.[1] While cancer treatment aims to slow cancer progression, it also accelerates aging, and speeds the development of frailty.[2] Cancer treatments, such as chemotherapy, can cause molecular and cellular changes that lead to cell senescence and cause fatigue, inactivity, weakness, and slowness, resulting in frailty.[3] We and others have reported that adult cancer survivors receiving adjuvant cancer treatment have significantly higher rates of frailty than their peers not receiving systemic cancer treatment.[4–6] Frailty increases the risk of disability, falls, hospitalization, and death in both non-cancer and cancer populations.[7–13] Among participants in the Women’s Health Initiative diagnosed with cancer, frailty significantly increased after diagnosis and sustained or worsening frailty was associated with a 22%−25% higher mortality risk than women whose frailty status improved.[6] Similarly, in a cohort of cancer survivors aged 60 + enrolled in the third NHANES, post-diagnosis frailty had a 1.8 to 2.8-fold higher risk of premature mortality compared to non-frail survivors.[14]

Frailty is a dynamic state and can worsen in response to illness, aging, and/or stressors but can also potentially reverse with intervention. Since frailty is a multi-component syndrome, optimal interventions should target more than one component to achieve a dose sufficient to reduce clinical risk. Exercise has multiple benefits, and it has been shown to reduce frailty and improve individual frailty components in older adults without cancer.[15, 16] In a network analysis of trials consisting of single or combined interventions to reduce frailty in older adults, physical activity was the only intervention that significantly reduced frailty compared to controls (standardized mean difference: − 0.92, 95% CI − 1.55, − 0.29), out of other interventions such as nutrition, home modification or prehabilitation.[17] An exercise-based strategy holds strong potential for reversing frailty in people with cancer; however, since frailty components are criteria-based measures, an effective exercise program must change multiple components to a large enough degree to shift frailty. In 2019, the American College of Sports Medicine (ACSM) exercise guidelines for cancer survivors issued evidence-based exercise prescriptions of aerobic and/or resistance training for several cancer-related health outcomes, including fatigue and physical functioning,[18] while earlier guidelines reported that exercise could improve muscle strength in people with cancer.[19] However, evidence remained insufficient for several other important outcomes, including frailty and falls. [18] Other types of exercise not covered in the ACSM guidelines, such as tai chi[20] or yoga, have shown reductions in fatigue and improvements in muscle strength/endurance and physical activity in people with cancer and could also have the potential to reduce frailty and a less strenuous exercise option.[20, 21] Despite the strong potential for exercise to reduce frailty in people with cancer, there are no sufficiently large randomized controlled trials of exercise with frailty as an outcome.

The GET FIT (Group Exercise Training for Functional Improvement after Treatment) trial was designed to compare the relative efficacy of two distinct types of prescribed exercise interventions, strength training versus tai ji quan, to reduce falls in older, postmenopausal women who had previously received chemotherapy.[22] Since frailty is a precursor to falls and since strength training and tai ji quan may improve frailty components, we had an opportunity to conduct a secondary data analysis to explore the potential additional benefits of both exercise modalities on frailty in our study sample at risk for accelerated aging. Thus, our primary aim was to determine whether or not strength training and/or tai ji quan would improve the odds of being less frail or pre-frail compared to the stretching control group, with a secondary aim to determine whether and which of each of the frailty criteria were improved by strength training and/or tai ji quan compared to the stretching control group. We also aimed to explore which demographic, clinical and/or behavioral characteristics describe women who reduced frailty status in response to strength training or tai ji quan compared to women who did not.

We hypothesized that strength training and tai ji quan would each reduce frailty but could differ in terms of which frailty components were improved compared to a control group (stretch and relaxation). We also hypothesized that women who might be more susceptible to being frail based on baseline demographic, health, and clinical characteristics (i.e., older age, poorer health) would be more likely to benefit than other women.

## Methods

### Patient Population

The target population for GET FIT included women cancer survivors previously treated with chemotherapy. Inclusion criteria were a diagnosis of stage I-III cancer other than brain or spinal cord, chemotherapy completion ≥ 3 months before enrollment, 50–75 years of age, postmenopausal status, physically underactive (< 60 minutes/week of self-reported moderate-intensity exercise), ability to provide consent, and absence of contraindications to moderate-intensity exercise (NCT01635413). The published study protocol was IRB approved, and written informed consent was obtained from each participant.[22] Women were enrolled from 2013 to 2018. Patients were recruited using mailings sent by the Oregon State Cancer Registry and direct referrals from Oregon Health and Science University (OHSU) and community clinicians. Staff screened and scheduled eligible participants who provided consent at baseline.

### Study Design

This study was a single-blind, parallel-group, randomized controlled trial. Participants were randomly assigned in a 1:1:1 ratio to one of three groups: (1) strength training, (2) tai ji quan, or (3) stretch and relaxation (control). Women participated in their assigned, supervised group training (10–15 participants/group) for 6 months. Interventions were conducted in person at OHSU and community locations. Each participant received a sealed, opaque envelope containing her randomly assigned sequence number generated by a statistician (ND) using a random number generator in MS Excel after completion of baseline testing, including surveys, which ensured that measurement technicians responsible for data collection and coding were blinded to group allocation. Participants were not told whether or not their group represented the experimental or control groups.

### Interventions

Each group attended supervised 1-hour classes led by a certified exercise instructor twice weekly for 6 months. Physician clearance was obtained for every participant before starting classes.

#### Strength training.

This intervention protocol involved progressive lower-body strength training to improve neuromuscular function (strength, gait, and balance). This training program consisted of 1–3 sets of 8–10 exercises at a weight performed at 8–12 repetitions with a 1–2 minute rest between sets. Weighted vests and steps provided resistance, and starting intensity was tailored to each participant’s initial capability. Planned progression increases were 1–3% of body weight in weight vests per month, up to a target of 15% by 6 months, depending on tolerance.

#### Tai ji quan.

The intervention protocol was a set of 8 purposeful and functional movement forms blended with practice variation and therapeutic moves designed to challenge balance control and train gait patterns, including movement exercises such as ankle sways, displacement of the body’s center of mass over the base of support, trunk rotation/flexion, coordinated eye-hand movements, and multi-directional stepping. Training was progressive, with the number of forms doubling and repetitions increasing (approximately 2–4 each month for the first 3 months) and the forms’ complexity gradually increasing thereafter.

#### Stretching and relaxation control.

We chose a placebo exercise group over a non-exercise control group since exercise is recommended as usual care for all cancer survivors. The protocol involved whole-body stretches from a seated or lying position to minimize weight-bearing forces that might increase muscle strength or postural control. Each session’s last 10–15 minutes focused on progressive neuromuscular relaxation exercises.

### Study Outcomes and Assessments

Baseline demographic and clinical data were assessed by self-report and height and weight by anthropometry (Table 1). Outcomes were measured at baseline, 3 (mid-intervention), and 6 months (post-intervention). The primary outcome for this analysis was a 4-criteria frailty measure, with secondary outcomes of individual frailty criteria.

### Frailty

We measured frailty using a 4-criteria model consisting of fatigue/exhaustion, slowness, weakness, and inactivity. We did not include a measure of unintended weight loss, part of the original Fried frailty phenotype,[12] because we did not have a comparable measure of this outcome in the GET FIT trial. Furthermore, GET FIT was not intended to preserve or reverse unintended weight or muscle mass loss; thus, it would be unexpected to improve this frailty component. We defined frailty categories similar to Fried, where the absence of any frailty criteria = robust, 1–2 = pre-frail, and 3–4 = frail. The measurement of frailty criteria from GET FIT is described in detail elsewhere,[22] while the cutoff for having each of the criteria is described next. We determined the number of criteria met at baseline from pre-intervention testing data and reassessed at 6 months from post-intervention data.

#### Fatigue/Exhaustion:

We measured fatigue/exhaustion using the response to either of two items on the Centers for Epidemiologic Depression scale: “I felt that everything I did was an effort” and “I could not get ‘going.’” Participants met the frailty criteria for fatigue/exhaustion if they selected “Occasionally or a moderate amount of the time (3–4 days)” or “Most or all of the time (5–7 days)” to either question, respectively.[12]

#### Weakness:

We measured weakness by time to complete five chair stands. To meet this criterion, we applied the cutoff of 12 seconds or longer, which is a clinical cutoff indicating increased fall risk among older adults.[23]

#### Slowness:

We measured slowness by the usual gait speed over 4 meters (m/s). Participants completed two walking trials, and we used the average of both trials, with a cutoff of < 0.9 m/s as the cutoff for meeting this criterion.[12]

#### Inactivity:

We measured inactivity by the CHAMPS physical activity questionnaire, using < 383 kcals/week spent in moderate-vigorous physical activities as the cutoff for meeting this criterion.[12]

### Statistical Analysis

Planned sample size for the original clinical trial was based on a primary outcome of fall rate and resulted in 456 participants (152 per group), accounting for 25% attrition. For this secondary data analysis, we limited the analytic sample to participants who provided both baseline and 6-month observations for all frailty measures (n = 386; 85% of the original sample), resulting in the following group sizes: tai ji quan (n = 124), strength training (n = 129), and stretching control (n = 133). For Aim 1, improvement in frailty phenotype was defined as moving from one frailty phenotype at baseline to a more favorable one (i.e., frail to pre-frail) after 6 months. Logistic regression was then used to predict improvement in frailty phenotype comparing strength training and tai ji quan to stretching control. For Aim 2, improvement in a single frailty criterion was defined as moving from meeting the cutoff at baseline to no longer meeting the cutoff at 6 months. Logistic regression was used to compare the odds of improvement in any one or more frailty criteria versus no change/worsening between strength training and tai ji quan versus stretching control. The probability of improving specific frailty criteria was also modeled by the number of frailty criteria present at baseline. In this way we could examine the impact of initial frailty status on the probability of improvement. For Aim 3, we defined a responder as a woman who improved in one or more frailty criteria from baseline to 6-months. We then compared responders and non-responders on baseline characteristics (age, BMI, comorbidities, initial frailty phenotype, cancer type [breast vs. other], and intervention adherence) using 2-sample t-tests and Pearson’s chi-squared tests of proportion. All analyses were completed in R[24] using two-tailed tests and alpha = 0.05.[25] Interpretation of results focused on measures of effect size (e.g., Odds Ratios) and 95% confidence intervals.

## Results

### Study Sample

Women in the analytical sample (mean age: 62.0 ± 6.4 years) had stage I or II disease (67%) breast cancer (71%), and were 5.7 years past chemotherapy completion (Table 1). The analytical group is nearly identical to the full GET FIT sample.[26] The average number of frailty components at baseline was 1.22 ± 1.04, with no significant differences across study groups for this nor any other baseline characteristic. Adherence to twice-weekly exercise sessions for this analytical sample averaged 74.7% + 18.5 of prescribed classes, similar to the Intent to Treat (ITT) sample for fall outcomes in the primary GET FIT analysis.

### Frailty Phenotype

At baseline, 110 women (28%) were considered robust, 223 pre-frail (58%) and 53 frail (14%). The proportions of women in each frailty phenotype did not significantly differ by assigned study group (Table 1).

**Figure 1:**
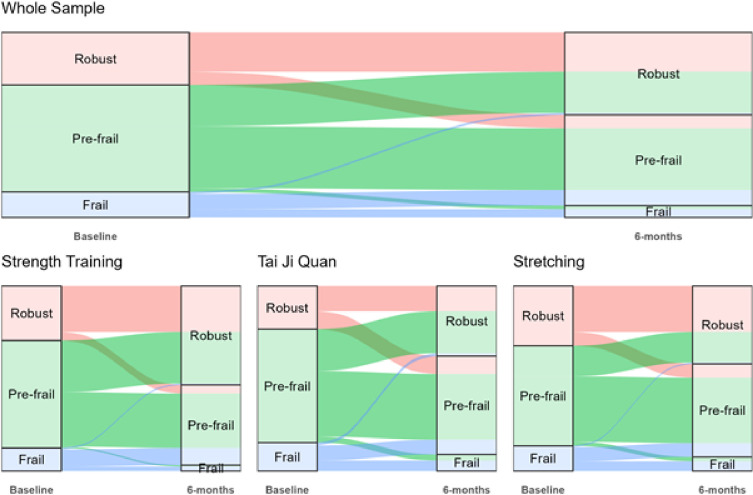
Sankey diagrams showing movement in frailty phenotypes (ribbons) from baseline to 6 months for the whole sample (top panel) and for each study group (bottom panel). Columns also illustrate the proportion of women in each frailty category at baseline (left-hand column) and at 6 months (right-hand column).

As shown in the Sankey diagrams, there was a movement across frailty phenotypes from baseline to 6 months (post-intervention) (Fig. 1). Visual inspection suggests that a higher proportion of women in the strength group moved from pre-frail to robust and from frail to pre-frail than other groups, with tai ji quan having slightly more favorable effects than stretching control. The odds of moving to a better frailty phenotype over 6 months in the strength training group was significantly higher as compared to the stretching control group (OR [95%CI]: 1.86 [1.09, 3.17]) (Table 2). The odds of moving to a better frailty phenotype in the tai ji quan group were also higher but not significantly different compared to stretching control (1.44 [0.84, 2.50]).

### Frailty Criteria

**Figure 2:**
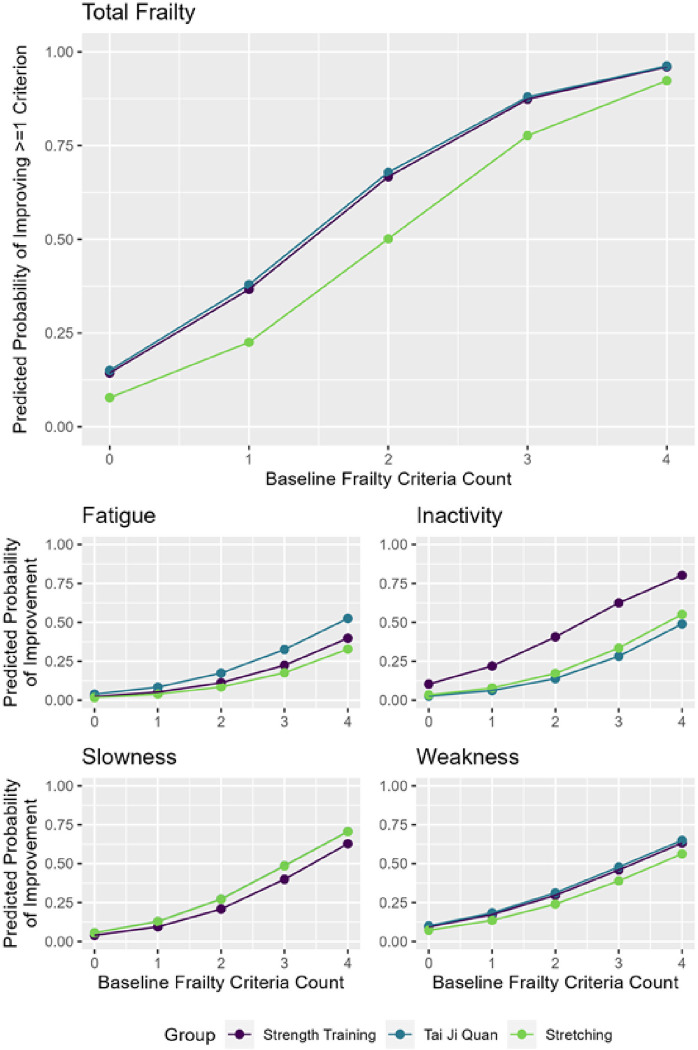
Predicted probability plots showing the likelihood that women within each study group improve in one or more frailty criteria (top panel) or in individual frailty criterion (bottom four panels) as a function of the number of frailty criteria present at baseline. Probability values range from 0–1.0, where 0 = 0% chance of improvement and 1.0 = 100% chance of improvement in the outcome.

When controlling for the number of frailty criteria at baseline, women in the strength training or tai ji quan group were twice as likely to improve in at least one frailty criteria (i.e., no longer meet the cutoff score) compared to stretching. Strength training (1.99 [1.10, 3.65]) and tai ji quan (OR 2.10 [1.16, 3.84]) were more likely to improve 1 or more frailty criteria compared to stretching control (Table 2). Among women who improved, 69% improved 1 criterion, 28% improved 2 criteria, and 3% improved 3 criteria. We also plotted the predicted probability for each study group to improve the overall frailty phenotype and individual frailty criteria by baseline frailty phenotype (Fig. 2). For the overall frailty phenotype, women who met four criteria were highly likely to improve in at least one criterion over 6 months, while women with fewer frailty criteria were more likely to improve if they were in the strength training or tai ji quan training groups. For fatigue/exhaustion, at any baseline level of frailty, women in tai ji quan were more likely to improve this component than women in other groups. For inactivity, at any baseline level of frailty, women who strength trained were more likely to improve this component than women in other groups. Women were more likely to improve for slowness (4m usual walk speed) and weakness (5-time sit-to-stand) as the number of frailty criteria they met at baseline increased, but group differences were negligible (Fig. 2). When examining individual frailty components, compared to stretching control (Table 2), tai ji quan tended to reduce fatigue enough to avoid this frailty criterion (OR: 2.26 [0.93, 5.93]). In contrast, strength training was more likely to reduce inactivity enough to avoid this criterion compared to controls (OR: 3.29 [1.65, 6.85]).

### Responders vs. Non-Responders

We compared several characteristics that might differentiate women more likely to reduce frailty in response to 6 months of strength training or tai ji quan, including age, BMI, comorbidities, initial frailty phenotype, cancer type (breast vs. other), and intervention adherence, independent of the assigned study group (Table 3). Women significantly more likely to reduce frailty levels over 6 months had higher BMI (BMI: 30.6 kg/m^2^ ± 5.6 kg/m^2^), higher Charlson comorbidity index (CCI) scores (CCI: 2.1 ± 1.6), and more frailty criteria (1.9 ± 0.9) at baseline than women who did not improve on one or more frailty components (BMI: 28.7 kg/m^2^ ± 6.5 kg/m^2^; CCI: 1.7 ± 1.5; and, frailty criteria: 0.8 ± 0.9).

## Discussion

In our secondary data analysis, we found evidence that both fall-prevention exercise training approaches from the GET FIT trial may effectively address frailty in older postmenopausal women treated with chemotherapy. Strength training benefited the most, leading to an 86% higher chance of reducing overall frailty levels and two-fold better odds of improving at least one frailty component than a seated stretching control group. Tai ji quan also showed a positive trend toward reducing frailty and was twice as likely to improve at least one criterion compared to controls. The benefits of strength training and tai ji quan were consistently better than stretching at any baseline level of frailty, except for the most frail, who were likely to improve from any study program. Women were more likely to benefit from the study programs had a higher BMI, comorbidity score, and frailty status before starting the study exercise programs compared to other women.

Our analysis is the first to show that supervised, group exercise training can reduce frailty in women cancer survivors experiencing accelerated aging most likely associated with prior chemotherapy treatment. Frailty is a dynamic state, and over 6 months, we observed movement within our sample where women could move from pre-frail or frail to less frail states. Strength training reduced overall frailty more than other groups, which is consistent with the established benefits of this modality on multiple frailty constructs (i.e., muscle strength, fatigue, and physical activity) in people with cancer.[18, 27, 28] Our program targeted the lower body and focused on functional movement patterns because the original aim was to decrease falls, thus this specific type of strength training was also specific enough to target physical frailty criteria. Tai ji quan targets neuromuscular function differently than strength training emphasizing postural control through slow choreographed movements that challenge balance and stability. Though frailty levels improved among women in tai ji quan, changes did not reach statistical significance. In studies of tai ji quan in people with and without cancer, this modality can reduce fatigue, slowness, and weakness and increase overall activity levels.[20, 21] A higher frequency or longer duration of tai ji quan may be needed to improve frailty components enough to shift the overall phenotype.

Women engaged in strength training or tai ji quan were twice as likely to improve in at least one frailty criterion as compared to women who participated in seated stretching. However, the modalities changed individual criteria differently. Women in the strength training group were 3.5 times more likely to increase their self-report physical activity enough to no longer meet the frailty criteria of inactivity. Participation in strength training can improve self-efficacy for exercise and exercise self-confidence thus, women may have felt more capable of engaging in more physical activity in their daily lives.[29, 30] Since we focused our strength training program on functional movements it is also possible that women had more muscular strength and endurance to engage in more frequent and/or longer periods of physical activity. Conversely, women in tai ji quan were twice as likely to report levels of fatigue that no longer met the criterion for frailty. Our tai ji quan program consisted of eight movement forms and additional therapeutic movements all done continuously in a standing position for up to 60 minutes, likely leading to improvements in muscular endurance and work capacity that can reduce fatigue.[20, 21, 31] Neither strength training nor tai ji quan significantly improved the other physical frailty criteria enough to significantly improve frailty status for these outcomes. This finding was a bit surprising since strength training significantly increased maximal muscle strength in the primary analysis of GET FIT, while tai ji quan improved postural stability. In this subsample, fewer women within each study group may have met these frailty criteria at baseline and thus had less room for improvement, thereby making it less likely to see group differences. However, since some women did improve in two or more frailty criteria, we suspect that there was some improvement in these physical frailty criteria among those with lower functioning at baseline. Future studies that aim to reduce frailty should carefully consider whether or how the target sample is defined based on initial frailty so that ceiling effects are limited.

We also took the opportunity to identify characteristics of women most likely to improve in the frailty criteria in response to the experimental exercise programs. We found that women with higher BMI, more comorbidities and more frailty were more likely to improve in one or more frailty criteria compared to women who were generally healthier. Other than receipt of chemotherapy, we did not specifically identify a target sample with known frailty or frailty risk for the original study. Yet, our sample had rates of frailty on par or higher than other studies in cancer survivors, because it included all women who are known to be at higher risk of frailty for a given age.[4, 6, 14] Obesity and comorbidities are associated with frailty in people with cancer,[4, 5, 32] and our “responder” group also had higher levels of frailty at baseline. Hence, this group may inadvertently have become a reasonable target sample, who according to the principle of initial values, had the greatest room and need for improvement. Notably, the higher BMI consistent with obesity, higher comorbidities, and more frailty did not adversely impact the ability of these women to participate and respond to regular, structured group exercise training. Future studies may wish to consider these characteristics when identifying an appropriate target sample to study frailty.

Our study is among the first to determine whether exercise can reduce frailty in people with cancer and begins to contribute to the developing exercise prescriptions for frailty for future exercise guidelines for cancer survivors. There are some additional strengths. One advantage of conducting a secondary data analysis on a large, well-powered, and complex behavioral intervention is that additional outcomes of interest can be examined more quickly and with substantial cost savings than performing a new trial. Since GET FIT was aimed at falls as a primary outcome and frailty is a precursor to falls, the methods used in the original study are well aligned with an analysis to examine frailty. The experimental interventions in GET FIT were selected because they were well-established fall prevention approaches in non-cancer populations and were potentially accessible in community settings. Accordingly, the same advantages of testing these programs apply in this analysis focused on frailty. The GET FIT study was sufficiently powered for falls, and adherence and compliance to all study exercise programs were high; consequently, the study fidelity for this analysis was strong.

Our study also has limitations. GET FIT was not designed *a priori* to address frailty, a multi-component syndrome, and it is possible that a more targeted and/or multi-component intervention would have greater effects on frailty than a single modality. The choice of our stretching control group for GET FIT was selected because a seated flexibility program was expected to have little effect on falls and reduce unequal attention and/or attrition with a usual care control. As seen in the probability analysis, even low-intensity activity such as seated stretching may have some benefit in women who are more frail; thus, we may have underestimated the benefit of strength training and tai ji quan compared to no exercise. Our sample was limited to women under 75 years of age since we set the original inclusion criteria for GET FIT to minimize the influence of other comorbidities and advanced age on fall. Therefore, we cannot be certain our findings generalize to much older women, who may have the highest prevalence of frailty. Nonetheless, three-quarters of our sample between the ages of 50–75 years met criteria for pre-frailty and frailty which corroborates our prior findings of early onset frailty in women breast cancer survivors, and reinforces the concept of accelerated aging in this population. Clinicians should be aware that even their non-elderly patients may be at risk for frailty or have frailty and could benefit from regular structured exercise programs. Finally, we did not include a measure of unintended weight loss or sarcopenia in our frailty measure. Still, given the high levels of overweight and obesity in our sample, we suspect this criterion had little influence on our findings.

This analysis furthers our understanding of whether and how two well-established exercise modalities could reduce frailty related to chemotherapy. By 2040, the proportion of cancer survivors over 65 years old will rise to 73%, with most surviving 5 years or longer.[1] Within just 5 years the number of older survivors in the U.S. alone will reach 14 million. Thus, there remains an urgent need to develop low-cost, accessible, efficacious, and appropriate interventions to reduce frailty and its adverse consequences in older cancer survivors.[33] Our analysis suggests that two types of exercise that target neuromuscular functioning in different ways can address the problem of frailty, and in turn offer different exercise options for older cancer survivors. By reducing frailty, exercise may interrupt the downward trajectory toward disability and dependence and, in turn, potentially delay or avoid hospitalization and premature death. Future studies designed to reduce frailty in people with cancer can build on these promising findings to refine the optimal modalities or combinations thereof, and the dose, timing and at-risk populations in order to reduce frailty associated with the combined effects of aging and cancer.

## Figures and Tables

**Table 1:** Baseline characteristics for the whole sample and by assigned study group.

Characteristic	Whole Sample		Strength Training	Tai Ji Quan	Flexibility	p-value
	(N = 386)		(n = 129)	(n = 124)	(n = 133)	
	Mean (SD) or %	Range	Mean (SD) or %	Mean (SD) or %	Mean (SD) or %	
Age (years)	62.0 (6.4)	50.0–75.0	62.8 (6.2)	62.0 (6.4)	61.2 (6.5)	.105
Race						
White	90.7%		91.5%	90.3%	90.2%	.733^a^
Black	1.6%		1.6%	0.8%	2.3%	
Asian	2.9%		2.3%	3.2%	3.0%	
Native American / Native Alaskan	0.8%		1.6%	0.8%	0.0%	
Native Hawaiian / Pacific Islander	0.3%		0.0%	0.0%	0.8%	
More than one race	3.1 %		1.6%	4.0%	3.8%	
Hispanic ethnicity	1.3%		1.6%	81.0%	1.5%	1.000
Body Mass Index (kg/m^2^)	29.5 (6.6)	17.0–53.3	29.6 (5.8)	30.0 (7.2)	28.9 (6.7)	.378
Charlson Comorbidity Index	1.9 (1.5)	0.0–12.0	1.9 (1.7)	1.8 (1.5)	1.9 (1.4)	.739
Initial frailty status						
Robust	28.5%		29.5%	23.4%	32.3%	.586
Pre-frail	57.8%		58.1%	61.3%	54.1%	
Frail	13.7%		12.4%	15.3%	13.5%	
Frailty criteria count (0–4)	1.2 (1.0)	0.0–4.0	1.2 (1.0)	1.4 (1.1)	1.2 (1.1)	.182
Cancer type						
Breast	71.0%		69.0%	74.2%	69.9%	.817^b^
Cervical	1.0%		1.6%	0.0%	1.5%	
Colon	6.5%		7.8%	5.7%	6.0%	
Liver	.3%		0.0%	0.8%	0.0%	
Lung	2.3%		3.1%	2.4%	1.5%	
Lymphoma	4.2%		3.1%	3.2%	6.0%	
Ovarian	6.0%		6.2%	5.7%	6.0%	
Pancreatic	0.3%		0.0%	0.8%	0.0%	
Urinary/bladder	0.3%		0.8%	0.0%	0.0%	
Uterine	2.6%		4.7%	1.6%	1.5%	
Other	5.7%		3.9%	5.7%	7.5%	
Cancer stage						
I	28.0%		32.6%	24.2%	27.1%	.424^c^
II	39.4%		33.3%	43.6%	41.4%	
III	26.4%		28.7%	25.0%	25.6%	
Not staged	1.0%		0.8%	1.6%	0.8%	
Donť know	5.2%		4.7%	5.7%	5.3%	
Time since diagnosis (years)	5.7 (4.2)	.6–18.8	5.6 (4.3)	6.0 (4.0)	5.5 (4.3)	
Time since chemotherapy completion (years)	5.1 (4.2)	.3–18.3	5.0 (4.3)	5.1 (3.9)	5.0 (4.3)	

a p-value compares white to all other races combined.

b p-value compares breast to all other cancer types.

c p-value compares stages I, II, and III only.

**Table 2:** Odds ratios of improving frailty criteria compared to stretching control group. Higher odds ratios indicate an increased likelihood of improving on a criterion.

Frailty Criteria	Strength Training	p-value	Tai Ji Quan	p-value
	OR (95% CI)		OR (95% CI)	
Improvement by at least one criterion	1.99 (1.10, 3.65)	.025	2.10 (1.16, 3.84	.015
Exhaustion	1.35 (.50, 3.78)	.553	2.26 (.93, 5.93)	.081
Inactivity	3.29 (1.65, 6.85)	.001	0.78 (.34, 1.75)	.543
Slowness	0.71 (.34, 1.44)	.340	1.00 (.51,1.93)	.992
Weakness	1.33 (.69, 2.61)	.395	1.44 (.75, 2.79)	.275

Note: models control for the number of frailty criteria present at baseline

**Table 3:** Baseline demographic and clinical characteristics and exercise session attendance between participants who improved at least one frailty criterion and those who did not improve or worsened over the 6-month intervention period.

Characteristic	Improved	Did Not Improve	p-value
	(n = 122)	(n = 139)	
	Mean (SD) or %	Mean (SD) or %	
Baseline number of frailty components	1.9 (0.9)	0.8 (0.9)	<0.001
Baseline frailty status			
Robust	0.0%	48.2%	<0.001
Pre-frail	77.2%	45.3%	
Frail	22.8%	6.5%	
Age (years)	62.5 (6.8)	62.2 (5.9)	0.630
Body Mass Index (kg/m^2^)	31.1 (6.7)	28.7 (6.1)	0.004
Charlson Comorbidity Index	2.1 (1.7)	1.6 (1.5)	0.018
Cancer type			
Breast	70.2%	72.7%	0.767
Cervical	1.8%	0.0%	
Colon	7.0%	6.5%	
Liver	0.9%	0.0%	
Lung	3.5%	2.2%	
Lymphoma	3.5%	2.9%	
Ovarian	6.1 %	5.8%	
Pancreatic	0.9%	0.0%	
Urinary/bladder	0.0%	0.7%	
Uterine	3.5%	2.9%	
Other	2.6%	6.5%	
Cancer stage			
I	29.0%	28.1%	0.168
II	35.1%	41.0%	
III	29.8%	24.5%	
Not staged	1.8%	0.7%	
Donť know	4.4%	5.8%	
Time since diagnosis (years)	5.4 (3.8)	6.0 (4.4)	0.243
Time since chemotherapy completion (years)	4.8 (3.7)	5.4 (4.4)	0.158
Class attendance (%)	75.7 (18.2)	75.4 (16.1)	0.890

a p-value compares breast to all other cancer diagnoses.
